# Preliminary Insights into the Phylogeography of Six Aquatic Hyphomycete Species

**DOI:** 10.1371/journal.pone.0045289

**Published:** 2012-09-18

**Authors:** Sofia Duarte, Sahadevan Seena, Felix Bärlocher, Fernanda Cássio, Cláudia Pascoal

**Affiliations:** 1 Centre of Molecular and Environmental Biology (CBMA), Department of Biology, University of Minho, Campus de Gualtar, Braga, Portugal; 2 Department of Biology, Mount Allison University, Sackville, New Brunswick, Canada; University of California Riverside, United States of America

## Abstract

Aquatic hyphomycetes occur worldwide on a wide range of plant substrates decomposing in freshwaters, and are known to play a key role in organic matter turnover. The presumed worldwide distribution of many aquatic hyphomycete species has been based on morphology-based taxonomy and identification, which may overlook cryptic species, and mask global-scale biogeographical patterns. This might be circumvented by using DNA sequence data. The internal transcribed spacer (ITS) region from rDNA was recently designated as the most suitable barcode for fungal identification. In this study, we generated ITS barcodes of 130 isolates belonging to 6 aquatic hyphomycete species (*Anguillospora filiformis*, *Flagellospora penicillioides*, *Geniculospora grandis*, *Lunulospora curvula*, *Tetrachaetum elegans* and *Tricladium chaetocladium*), and collected from streams of Southwest Europe (86 isolates) and East Australia (44 isolates). European and Australian populations of 4 species (*A. filiformis*, *F. penicillioides*, *G. grandis* and *T. elegans*) grouped into different clades, and molecular diversity indices supported significant differentiation. Continents did not share haplotypes, except for *T. chaetocladium*. Overall results show substantial population diversity for all tested species and suggests that the biogeography of aquatic hyphomycetes may be species-specific.

## Introduction

Most large eukaryotic plants and animal species have relatively narrow geographic distributions. By contrast, it has long been assumed that prokaryotic microorganisms occur essentially everywhere and will grow and reproduce whenever conditions are suitable: “everything is everywhere, but the environment selects” [1]. Fenchel and Finlay [2] and Finlay and Fenchel [3] extended the assumption of global distribution to eukaryotic microorganisms. They postulated a transition from ubiquity to biogeography at a body size between 1 and 10 mm. Many morphospecies (taxonomy based on morphological differences) of small aquatic animals and protists are indeed very widely distributed. However, molecular genetic evidence has shown that reliance on morphological data inflates the range of geographical distribution of many microorganisms, including prokaryotes, diatoms and protists [4]. Taylor et al. [5] reached the same conclusion concerning fungi. For instance, strains of *Neurospora discreta* were resolved into several distinct phylogenetic species with different geographic distributions [6], [7]. The genus *Lentinula* was thought to have 4 morphologically distinct species [8], but molecular analyses revealed that only 1 of the 4 was a single phylogenetic species. Each of the other 3 contained 2 phylogenetic species, all with more restricted geographic distributions [9]. Among fungi, *Aspergillus fumigatus* seems to be an exception. Even though the single morphospecies was split into two phylospecies, both exhibited global distribution [10].

Aquatic hyphomycetes occur worldwide on a wide range of plant substrates, such as leaves and wood, decomposing in freshwaters, and are known to play a key role in organic matter turnover [11], [12]. Phylogenetically, most species are affiliated with the Ascomycota [13]. Historically, their classification has been based on the morphology and development of conidia (asexually produced spores). Their predominantly tetraradiate or sigmoid shapes suggest convergent evolution due to the common need for attachment to a suitable substrate in flowing water [14]. Molecular data [15] support and extend the phylogenetic heterogeneity suggested by morphologies of the teleomorphs (the sexual reproductive stages) [16].

Many species of aquatic hyphomycetes appear to be cosmopolitan; others are more common in certain climatic zones regardless of longitude. Wood-Eggenschwiler and Bärlocher [17] concluded that species occurrence is rarely restricted by geological barriers or by distance. On a worldwide scale, temperature, together with its influence on vegetation has been suggested as the main factor determining their distribution. The observation of identical morphospecies on geologically young islands far from mainlands, such as the Hawaiian Islands [18], suggests efficient long-distance transport of viable inocula. However, these conclusions are based on morphospecies, which ignores cryptic species whose characterization requires molecular data. In the few phylogenetic studies on this group of fungi no conclusions concerning biogeography were drawn due to the lack of isolates from diverse and distant geographic areas [15], [19], [20], [21], [22]. By chance, in a study conducted by Seena et al. [23] the genotypes of *Articulospora tetracladia* were found to be geographically widespread with the exception of Malaysian isolates. In the largest study to date, Anderson and Shearer [24] observed high genotypic diversity among 391 isolates of *Tetracladium marchalianum* from rivers in Illinois and Wisconsin. Stable genetic differentiation was only observed between the most distant rivers (∼ 450 km). Analysis of partial β-tubulin sequence data suggested that all isolates belonged to a single species, and did not support the existence of cryptic species within *T. marchalianum*.

To provide further insight into the biogeography of aquatic hyphomycetes, we analyzed the internal transcribed spacer (ITS) sequences of 6 species isolated from streams in Southwest Europe and East Australia. We hypothesized that strains of these 6 morphospecies would exhibit very distinct biogeographic patterns due to the distance between the two continents and the lack of any landbridge. Although little is known about the evolutionary origins of aquatic hyphomycetes, freshwater Ascomycota are believed to have originated about 390 million years ago [25], [26]. Continental drift separated Australia from what was left of Pangea ca. 130 million years ago; in the absence of frequent exchange between the two continents, this should provide ample time for genetic (and morphological) differentiation. We selected the ITS region, which shows considerable variation among and within aquatic hyphomycete species [21], [22], [23] and was recently declared the most suitable barcode for fungal species (http://www.ecbol.org). The selected taxa (*Anguillospora filiformis*, *Flagellospora penicillioides*, *Geniculospora grandis*, *Lunulospora curvula*, *Tetrachaetum elegans* and *Tricladium chaetocladium*) are commonly found on decomposing plant-litter in freshwaters [27], [28], [29].

## Materials and Methods

### Dataset

The dataset consists of 130 ITS sequences belonging to 6 aquatic hyphomycete species, *A. filiformis*, *F. penicillioides*, *G. grandis*, *L. curvula*, *T. elegans* and *T. chaetocladium*, sampled from streams of Europe (86 isolates) and Australia (44 isolates) between 2009 and 2011 and separated by ca. 18000 km. The European isolates originated in continental Portugal (58), the Azores archipelago (7), Spain (19) and Italy (2). All isolates got in this study were deposited in the culture collection of the Centre of Molecular and Environmental Biology (CBMA), Department of Biology of the University of Minho prior to sequencing. Eighteen ITS sequences of the Portuguese isolates were obtained in a previous study [22] and had already been deposited in the NCBI. Table S1 shows an overview of all sequences used in our analyses.

### Sampling, Isolation and Culture Conditions

Isolates are maintained in the culture collection of the Centre of Molecular and Environmental Biology (CBMA), Department of Biology of the University of Minho. The methodology of fungal isolation is described in Pascoal et al. [29]. All cultures were grown at room temperature on 1% malt agar extract during ca. 15 days before DNA extraction.

### DNA Analyses

DNA was extracted with the MoBio Ultraclean Soil DNA Isolation kit according to the manufacturer’s instructions and stored at −20°C. For PCR reactions, 14 µL of Accuzyme mix (2x) (Bioline), ITS1 and ITS4 primers (1.6 µM) [30], MgCl_2_ (3 mM) and 2 µL of DNA (1–10 ng µL^−1^) were used in a final volume of 25 µL. PCR reactions were carried out in a Doppio thermocycler (VWR) as follows: 1) initial denaturation for 2 minutes at 94°C; 2) 40 cycles of denaturation for 45 seconds at 94°C; annealing for 45 seconds at 55°C and extension for 1 minute and 30 seconds at 72°C and 3) final elongation for 10 minutes at 72°C. The PCR products were run on a 2% agarose gel at 80V for 45 minutes to check the presence of the desired band. The PCR products were cleaned with a PureLink™ PCR purification Kit according to the manufacturer’s instructions (Invitrogen) and DNA concentration was checked with a nanodrop instrument (Spectrophotometer ND-1000, VWR). The amplicons were sequenced at StabVida (Oeiras, Portugal) using ITS1 and ITS4 primers [30].

### Data Analyses

Consensus sequences of ITS region were obtained with CodonCode Aligner 2.0.6 (Codon Co., USA). Sequences were aligned using ClustalW [31], divergence was analyzed using Kimura 2-parameter (K2P) distance [32] and dendograms were generated with Neighbour-joining (NJ) method [33], using MEGA4 software [34]. Branch support was assessed with bootstrap analysis (1000 replicates) [35]. The ITS sequence of *Articulospora tetracladia* UMB-014.00 (GQ411288) from GenBank was used to root the trees. Sequence data obtained during this study were deposited in GenBank (Table S1).

Standard indices of molecular diversity, namely theta S (*θ_S_*) and theta pi (*θ_π_*), and pairwise *F_st_* values were obtained using Arlequin 3.5.1.2 [36]. Theta (*θ*) represents the distribution of variation within or among populations when samples are considered to represent characteristics of the larger group from which they are sampled. Theta S exhibits the infinite site equilibrium relationship between polymorphic sites, sample size and *θ*, for non-recombining DNA sample, while *θ_π_* estimates the infinite site equilibrium relationship between the mean number of pairwise differences and *θ* [37], [38].

## Results

The phylogeography of 130 isolates belonging to 6 aquatic hyphomycete species was inferred from ITS sequences. Total sequence length varied from 513 to 577 bp (*A. filiformis*, 513–514 bp; *T. elegans*, 513–520 bp; *T. chaetocladium*, 577 bp; Table 1). Isolates of *T. elegans* showed the highest variation in sequence length, while those of *G. grandis* (519 bp) and *T. chaetocladium* did not vary. *Anguillospora filiformis* showed the highest nucleotide percentage of A+T, while *L. curvula* and *T. chaetocladium* showed the highest percentage of G+C (Table 1).

**Table 1:** Sequence length, nucleotide composition, number of isolates per country, and % divergence between and within countries of ITS sequences for each of the 6 aquatic hyphomycete species.

		AF	FP	GG	LC	TE	TC
Sequence length (bp)							
		513–514	517–519	519	520–524	513–520	577
Nucleotide composition							
A (%)		23.9–24.2	22.8–23.1	22.2–22.7	22.3–22.5	23.1–23.6	19.8–19.9
T (%)		26.9–27.1	24.1–24.6	24.3–25.2	21.9–22.6	25.9–26.7	25.1–25.5
G (%)		24.3–24.4	24.3–24.8	26.4–27.0	26.2–26.7	24.2–25.1	26.5
C (%)		24.4–24.7	27.8–28.3	25.6–26.6	28.6–29.0	23.1–23.6	28.2–28.6
Country (n° of isolates)							
		Pt (12)	Pt (13)	Pt (2)	Pt (10)	Pt (10)	Pt (13)
		Aus (5)	Az (7)	Aus (5)	Aus (5)	Aus (5)	Aus (11)
			Aus (13)	Sp (3)	Sp (3)		Sp (2)
			Sp (11)				
			It (2)				
Average divergence (%)							
		0.2±0.1	0.8±0.2	0.9±0.3	0.2±0.1	2.0±0.4	0.1±0.1
Divergence within countries (%)							
		Pt:0.2±0.1	Pt:0	Pt:0	Pt:0	Pt:0	Pt:0.1±0.1
		Aus:0	Sp:0.3±0.1	Sp:0	Aus:0.5±0.2	Aus:0	Aus:0
			Aus:0	Aus:0	Sp:0.1±0.1		Sp:0
			Az:0.3±0.2				
			It:0				
Divergence between countries (%)	Distance Between Countries (Km)						
Aus vs Az	18899	−	0.3±0.2	−	−	−	−
Aus vs It	16311	−	0.4±0.3	−	−	−	−
Aus vs Pt	18054	0.2±0.1	1.8±0.6	1.6±0.5	0.3±0.1	4.2±0.9	0.1±0.0
Aus vs Sp	17671	−	0.3±0.2	1.6±0.5	0.3±0.1	−	0.2±0.2
Az vs It	2774	−	0.3±0.2	−	−	−	−
Az vs Pt	1185	−	1.8±0.6	−	−	−	−
Az vs Sp	1452	−	0.3±0.2	−	−	−	−
It vs Pt	1752	−	1.6±0.6	−	−	−	−
It vs Sp	1360	−	0.2±0.1	−	−	−	−
Pt vs Sp	423	−	1.7±0.6	0	0.1±0.1	−	0.2±0.1

Results are based on pairwise comparisons. Standard error estimate(s) were obtained by bootstrap (1000 replicates). Analyses were conducted using the Kimura 2-parameter method in MEGA4. All positions containing alignment gaps and missing data were eliminated in pairwise sequence comparisons (pairwise deletion option). AF, *A. filiformis*; FP, *F. penicillioides*; GG, *G. grandis*; LC, *L. curvula*; TE, *T. elegans* and TC, *T. chaetocladium*. Pt, Portugal; Az, Portugal (Azores); Sp, Spain; It, Italy; Aus, Australia.

**Figure 1:**
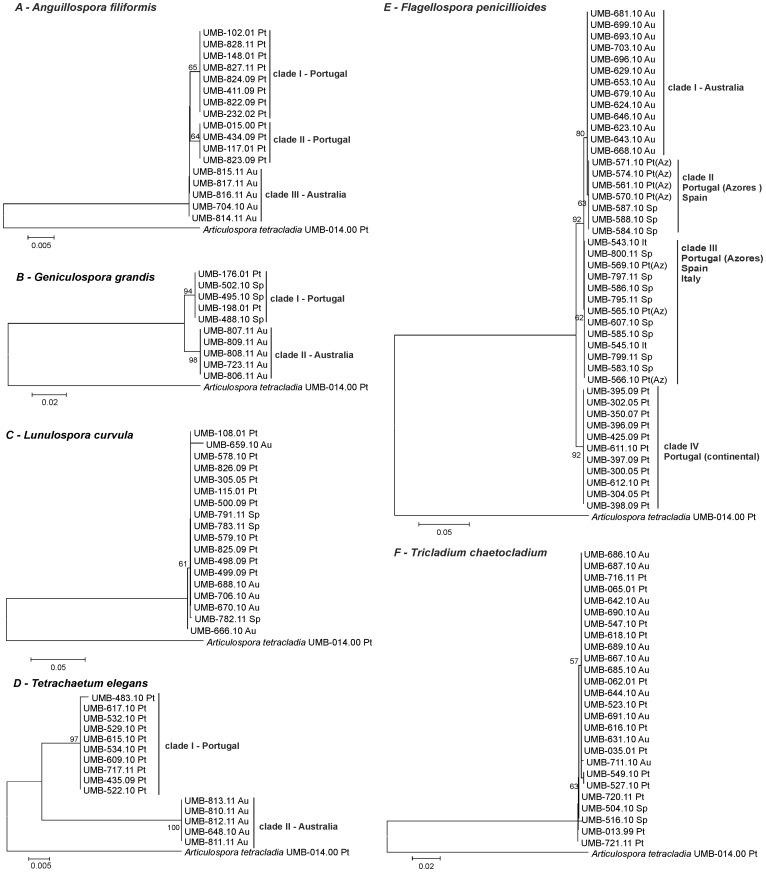
Neighbour joining trees based on ITS sequences. Neighbour joining trees based on ITS sequences using Kimura 2-parameter distances for *A. filiformis* (A), *G. grandis* (B), *L. curvula* (C), *T. elegans* (D), *F. penicillioides* (E) and *T. chaetocladium* (F); bootstrap values above 50% calculated from 1000 full heuristic replicates are shown at the nodes. Scale bar indicates one base change per 100 nucleotides. The sequence of *Articulospora tetracladia* UMB-014.00 (GQ411288) from GenBank was used to root the trees.

Except for *L. curvula* and *T. chaetocladium*, NJ trees supported considerable population diversity within fungal species (Fig. 1). Isolates of *A. filiformis*, *G. grandis* and *T. elegans* from Portugal and Australia clustered into distinct clades (Fig. 1A, 1B and 1D), while isolates of *L. curvula* and *T. chaetocladium* did not exhibit geographic cohesiveness (Fig. 1C and 1F). *Flagellospora penicillioides* yielded 4 geographic clades (Fig. 1E): clade I consisted of isolates from Australia, clade II and III included isolates from Spain, Italy and the Azores archipelago (Portuguese Islands) and clade IV isolates from continental Portugal.

The average evolutionary divergence was lowest for *T. chaetocladium* (0.1±0.1%) and greatest for *T. elegans* (2.0±0.4%). *Geniculospora grandis* and *T. elegans* did not exhibit any sequence divergence within countries, while maximum divergence was found among Australian *L. curvula* isolates (0.5±0.2%) (Table 1). Divergence between countries was lowest for European (Portugal and Spain) isolates of *G. grandis* (0%) and highest for isolates of *T. elegans* from Portugal and Australia (4.2±0.9%) (Table 1).

**Figure 2:**
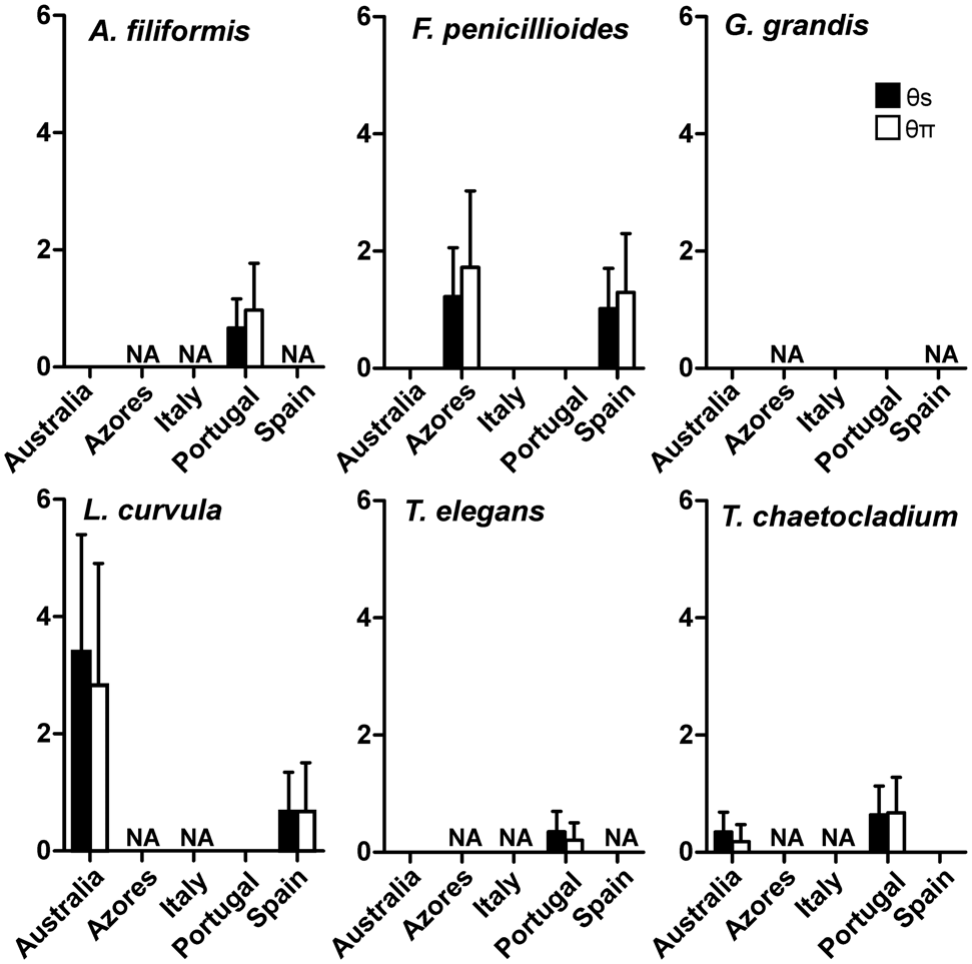
Molecular diversity indices, Theta S (*θ_S_*) and Theta pi (*θ_π_*) for each species within each country. Molecular diversity indices estimate the level of diversity existing within each country for each aquatic hyphomycete species. NA, not available.

**Figure 3:**
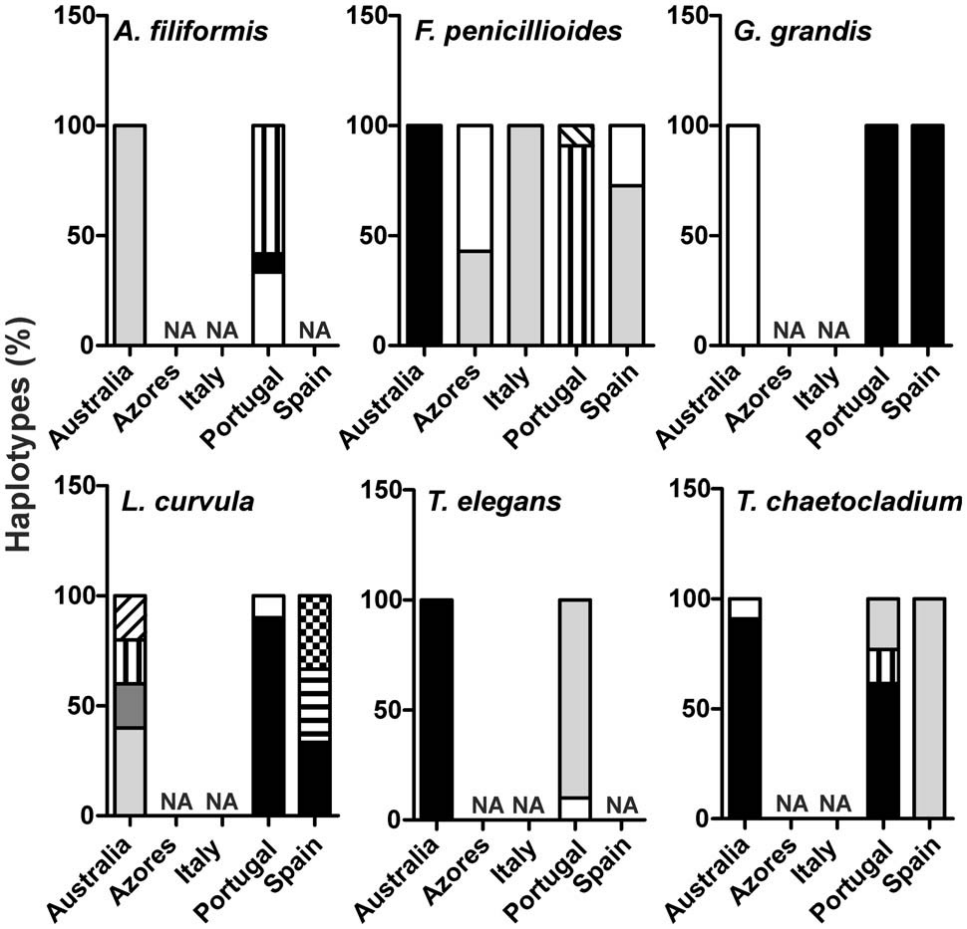
Haplotypes found for each aquatic hyphomycete species within each country. For each species one bar represents one country and similar patterns denote shared haplotypes. NA, not available.

Molecular diversity indices *θ_s_* and *θ_Π_* were higher for isolates from European than from Australian streams, except for *L. curvula* (Fig. 2). Australian isolates of *L. curvula* showed the highest molecular diversity indices within all species. On the other hand, *G. grandis* isolates did not exhibit any genetic variability within isolates of Portugal or any other country (Fig. 2). The number of haplotypes varied between 2 (*G. grandis*) and 8 (*L. curvula*) (Fig. 3). Australia did not share haplotypes with European countries except for *T. chaetocladium* (Fig. 3). On the other hand, European countries shared haplotypes of *F. penicilloides*, *G. grandis* and *L. curvula* (Fig. 3).

## Discussion

In the present study, we assessed the intraspecific diversity of 6 aquatic hyphomycete species by comparing strains from Southwest Europe and East Australia. Internal transcribed spacer regions had been used successfully to identify Portuguese isolates of *A. filiformis*, *F. penicillioides*, *G. grandis*, *L. curvula* and *T. chaetocladium* [22]. However, since the previous study was limited to fungal strains from a geographically restricted area (Northwest and Central Portugal), it did not allow any conclusions concerning cohesiveness between isolates from widely dispersed regions. Aquatic hyphomycete species might form genetic continua between a sequence of geographically distant connected locations. However, in sparsely populated or geographically isolated areas, genetically discrete taxa may be formed and assessed through the analysis of gene regions with high mutation rates (e.g. ITS). In the current study, we compared the ITS region in aquatic fungal isolates from Southwest Europe (continental Portugal, Portuguese Azores archipelago, Spain and Italy) and East Australia. The 6 selected species are common in all these geographically distant locations [27], [29]. Neighbour-Joining trees based on ITS showed substantial intrapopulation diversity for *A. filiformis*, *F. penicillioides*, *G. grandis* and *T.elegans*, and intraspecific clades were supported by bootstrap values generally ≥70%. By using computer simulations and a laboratory-generated phylogeny, Hillis and Bull [39] found that bootstrap values ≥70% usually correspond to a probability ≥95% that the corresponding clades are real. Four of the species (*A. filiformis*, *F. penicillioides*, *G. grandis* and *T. elegans*) displayed higher evolutionary divergence between isolates from different continents (Australia and Europe) than within continents. However, we should point out that some sampling sites belonging to the same continent were very close (e.g. sampling sites in Portugal and Spain for *G. grandis*), and thus these differences are expected to occur.

Although European and Australian isolates from *A. filiformis*, *F. penicillioides*, *G. grandis* and *T. elegans* grouped into different clades, there was no linear relationship between geographic distance and evolutionary divergence (p = 0.26, r^2^ = 0.065). For example, isolates of *F. penicillioides* from Portugal and Spain (ca. 450 km apart) diverged by 1.7%, while from Spain and Australia (ca. 18000 km apart) diverged only by 0.3%.

The maximum divergence of 4.2% was found between isolates of *T. elegans* from Australia and Portugal (ca. 18000 km apart), which exceeds the values reported for fungal intraspecific variability (0–3%) inferred from ITS sequences [40]. This may suggest the presence of cryptic species within the *T. elegans* species and deserves further investigation. Considerable intraspecific diversity was previously reported for *T. elegans* by random amplified polymorphic DNA (RAPD, [41]) and by amplified fragment length polymorphism (AFLP, [42]). However, no significant correlation was found between geographical and genetic distances, probably because streams were geographically close (0.5–18.5 km) [42].

Molecular diversity indices, *θ_s_* and *θ_Π_*, of isolates within a country were higher for Portugal than for Australia, except for *L. curvula*. On the other hand, *G. grandis* did not show any difference within isolates of the same country, but it should be noted that the number of isolates was low (2 to 5 per country contrasting with 3 to 10 per country for *L. curvula* or 2 to 13 per country for *F. penicillioides*, respectively). In addition, isolates of *G. grandis* were sampled at unique sites within each country, except for Portugal. The same pattern was found for Australian isolates of *A. filiformis* and *T. elegans* that were also sampled at unique sites. On the other hand, intraspecific diversity was greatest for Azores isolates of *F. penicillioides* that were sampled at two stream sites in S. Miguel Island.

European countries did not share any haplotypes with Australia, except for *T. chaetocladium*. It has been suggested that meiospores may be responsible for long-distance dispersal of aquatic hyphomycetes [43] and their production by *Hydrocina chaetocladia* (teleomorphic phase of *T. chaetocladium*) and *Nectria penicillioides* (teleomorph of *F. penicillioides*) have been documented [44]. Haplotypes of *F. penicillioides* from continental Portugal were also distinct from the ones in the Azores Islands, but the latter shared haplotypes with Spain and Italy. *Flagellospora penicillioides* has been reported as endophyte of plant roots [45], as all other species of this study with the exception of *T. elegans* [46]. Plant trading events in the 18^th^ and 19^th^ centuries and other historical or current commercial interactions [47], may have facilitated the genetic exchange between European countries and Azores. In some fungi, asexual reproductive structures (mitospores) have been reported to disperse within (e.g. continental Europe to Great Britain, [48]) and between continents (e.g. from South Africa to Australia, [49]; for other examples, see [5]). The relatively delicate conidia of aquatic hyphomycetes seem ill suited for longer distance dispersal. Nevertheless, in the current study, some widely distributed morphologically defined aquatic hyphomycete species were divided into genetically distinct populations with more narrow geographical distributions. A similar pattern was recently found for *Articulospora tetracladia*, whose genotypes appear to be widespread with the exception of Malaysian haplotypes [23]. Additional samples are needed to decide whether or not these differences are sufficiently consistent to define phylogenetic species. There is a growing concern that accelerating species loss may jeopardize ecosystem function and services. To evaluate this threat, we need to know intra- and interspecific variability of ecological functions and which species concept (morphospecies, phylospecies) is more relevant for capturing functional variability. An earlier review concluded that morphospecies of aquatic hyphomycetes have broadly overlapping functions [50]. On the other hand, conspecific strains, isolated from geographically close streams, can differ significantly in their ability to tolerate heavy metals and other pollutants (e.g., [51], [52]). It seems unlikely, though untested, that in these cases genetic differences have resulted in the formation of new phylospecies. Several examples from other fungal groups have been discussed by Taylor et al. [5]. The non-congruence of morphospecies and phylogenetic species is more pronounced in microorganisms than in macroorganisms. Taylor et al. [5] offer two non-exclusive reasons. Smaller organisms are morphologically less complex, providing less information to differentiate among forms. In addition, the rate of morphological change is slower for organisms with less elaborate development and fewer cells. The expected course of events in geographically widespread microbial species is therefore genetic differentiation, followed by reproductive and eventually morphological differentiation.

Our study suggests that there is no consistent inter- and intra-continental phylogeographic structure in aquatic fungi. The preliminary conclusion is that the biogeography or the extent of geographic distribution of aquatic hyphomycetes may be species-specific. Unfortunately, our sample size was small, since we relied on DNA from pure cultures that had been established from single conidia. This conventional approach is time-consuming and its success is greatly affected by chance. Analyses based on DNA extracted from individual spores potentially lower this hurdle [53], as will emerging next-generation sequencing techniques. Both nevertheless depend on a rich reference library of sequences from described fungal strains, which does not exist for aquatic hyphomycetes. Pyrosequencing potentially recovers all sequences in an environmental sample [54]. Access to these extensive datasets will vastly expand our capability of addressing phylogeography at a multi-species level, providing a better foundation to investigate biogeographic patterns in aquatic hyphomycetes.

## Supporting Information

Table S1 **Aquatic hyphomycete species, isolate reference, year of isolation, country of stream location, sampled substrate and Genbank accession number of sequenced isolates of the current study and those retrieved from NCBI.**
(DOCX)
