# Supplementary material for: Preliminary Insights into the Phylogeography of Six Aquatic Hyphomycete Species
Source: PLoS One. 2012 Sep 18;7(9):e45289. doi: 10.1371/journal.pone.0045289 (PMC3445450; doi:10.1371/journal.pone.0045289)
Supplement: Table S1 — Aquatic hyphomycete species, isolate reference, year of isolation, country of stream location, sampled substrate and Genbank accession number of sequenced isolates of the current study and those retrieved from NCBI. (DOCX) [file pone.0045289.s001.docx]

**Supporting information**

**Table S1. Aquatic hyphomycete species, isolate reference, year of isolation, country of stream location, sampled substrate and Genbank accession number of sequenced isolates of the current study and those retrieved from NCBI.**

The sampled substrates were: foam (F), leaves (L), stream water (SW) and twigs (T). Sampling sites are: Ave River (sites L1 and L7); Bellinger River (Ho, Le and Jo sites); Botão Stream (Bo); lake Bracciano (Br); Cávado River (Ca); Caldeiras Stream (Cas); Caldeirões Stream (Cao); Candal Stream (Can); Este River (sites E1 and E5); Estorãos River (Es); Hastings River (Ha); Lamas Stream (Lm); Madrid Stream (Ma); Moredun Creek (Mo); Mungay Creek (Mu); Never Never River (sites NN1, NN2); Onyar Stream tributary (On); Pelhe River (Pel); Petimão Stream (Pet); Souto stream (So); Tanha Stream (Ta); Vez Stream tributary (Ve) and Vigo Stream (Ve).

| Species | Isolate reference | Year | Stream location | Country | Sampled substrate | Genbank accession number |
| --- | --- | --- | --- | --- | --- | --- |
| *Anguillospora filiformis* Ingold | | | | | | |
|  | UMB-704.11 | 2011 | Mu (31º44’S 152º46’E) | Australia | L | JX089461 |
|  | UMB-814.11 | 2011 | Mu (31º44’S 152º46’E) | Australia | L | JX089462 |
|  | UMB-815.11 | 2011 | Mu (31º44’S 152º46’E) | Australia | L | JX089463 |
|  | UMB-816.11 | 2011 | Mu (31º44’S 152º46’E) | Australia | L | JX089464 |
|  | UMB-817.11 | 2011 | Mu (31º44’S 152º46’E) | Australia | L | JX089465 |
|  | UMB-015.00 | 2000 | L7 (41°20’N 8°31’W) | Portugal | SW | GQ411263^a^ |
|  | UMB-102.01 | 2001 | Pel (41°24’N 8°30’W) | Portugal | L | GQ411261^a^ |
|  | UMB-117.01 | 2001 | E5 (41°30’N 8°27’W) | Portugal | L | GQ411262^a^ |
|  | UMB-148.01 | 2001 | E5 (41°30’N 8°27’W) | Portugal | L | GQ411260^a^ |
|  | UMB-232.02 | 2002 | E1 (41°34’N 8°19’W) | Portugal | L | GQ411259^a^ |
|  | UMB-411.09 | 2009 | Es (41º77’N 8º62’W) | Portugal | L | JX089466 |
|  | UMB-434.09 | 2009 | Es (41º77’N 8º62’W) | Portugal | L | JX089467 |
|  | UMB-822.09 | 2009 | Es (41º77’N 8º62’W) | Portugal | L | JX089468 |
|  | UMB-823.09 | 2009 | Es (41º77’N 8º62’W) | Portugal | L | JX089469 |
|  | UMB-824.09 | 2009 | Es (41º77’N 8º62’W) | Portugal | L | JX089470 |
|  | UMB-827.11 | 2011 | E1 (41°34’N 8°19’W) | Portugal | L | JX089471 |
|  | UMB-828.11 | 2011 | E1 (41°34’N 8°19’W) | Portugal | L | JX089472 |
| *Flagellospora penicillioides* Ingold | | | | | | |
|  | UMB-623.10 | 2010 | NN2 (30º22’S 152º54’E) | Australia | L | JX089473 |
|  | UMB-624.10 | 2010 | NN2 (30º22’S 152º54’E) | Australia | L | JX089474 |
|  | UMB-629.10 | 2010 | Ho (30º14’S 153º9’E) | Australia | L | JX089475 |
|  | UMB-643.10 | 2010 | Jo (30º14’S 152º5’E) | Australia | L | JX089476 |
|  | UMB-646.10 | 2010 | Le (30º43’S 152º8’E) | Australia | L | JX089477 |
|  | UMB-653.10 | 2010 | NN1 (30º23’S 152º53’E) | Australia | L | JX089478 |
|  | UMB-668.10 | 2010 | Le (30º43’S 152º8’E) | Australia | L | JX089479 |
|  | UMB-679.10 | 2010 | Mo (30º08’S 151º3’E) | Australia | L | JX089480 |
|  | UMB-681.10 | 2010 | Mo (30º08’S 151º3’E) | Australia | L | JX089481 |
|  | UMB-693.10 | 2010 | Ha (30º56’S 151º58’E) | Australia | L | JX089482 |
|  | UMB-696.10 | 2010 | Ha (30º56’S 151º58’E) | Australia | L | JX089483 |
|  | UMB-699.10 | 2010 | Ha (30º56’S 151º58’E) | Australia | L | JX089484 |
|  | UMB-703.10 | 2010 | Mu (31º44’S 152º46’E) | Australia | L | JX089485 |
|  | UMB-561.10 | 2010 | Cas (37º79’N 25º45’W) | Azores | L | JX089486 |
|  | UMB-565.10 | 2010 | Cas (37º79’N 25º45’W) | Azores | L | JX089487 |
|  | UMB-566.10 | 2010 | Cas (37º79’N 25º45’W) | Azores | L | JX089488 |
|  | UMB-569.10 | 2010 | Cas (37º79’N 25º45’W) | Azores | L | JX089489 |
|  | UMB-570.10 | 2010 | Cas (37º79’N 25º45’W) | Azores | L | JX089490 |
|  | UMB-571.10 | 2010 | Cas (37º79’N 25º45’W) | Azores | L | JX089491 |
|  | UMB-574.10 | 2010 | Cao (37º50’N 25º16’W) | Azores | T | JX089492 |
|  | UMB-543.10 | 2010 | Br (42º07’N 12º13’E) | Italy | L | JX089493 |
|  | UMB-545.10 | 2010 | Br (42º07’N 12º13’E) | Italy | L | JX089494 |
|  | UMB-300.05 | 2005 | E1 (41°34’N 8°19’W) | Portugal | SW | GQ411324^a^ |
|  | UMB-302.05 | 2005 | E1 (41°34’N 8°19’W) | Portugal | SW | GQ411326^a^ |
|  | UMB-304.05 | 2005 | E1 (41°34’N 8°19’W) | Portugal | SW | GQ411325^a^ |
|  | UMB-350.07 | 2007 | Bo (40°18′N 8°23′W) | Portugal | L | GQ411323^a^ |
|  | UMB-395.09 | 2009 | So (41º31’N 8º17’W) | Portugal | L | JX089495 |
|  | UMB-396.09 | 2009 | So (41º31’N 8º17’W) | Portugal | L | JX089496 |
|  | UMB-397.09 | 2009 | So (41º31’N 8º17’W) | Portugal | L | JX089497 |
|  | UMB-398.09 | 2009 | So (41º31’N 8º17’W) | Portugal | L | JX089498 |
|  | UMB-425.09 | 2009 | Ve (41º99’N 8º33’W) | Portugal | L | JX089499 |
|  | UMB-611.10 | 2010 | Pet (41º48’N 8º01’W) | Portugal | L | JX089500 |
|  | UMB-612.10 | 2010 | Pet (41º48’N 8º01’W) | Portugal | L | JX089501 |
|  | UMB-583.10 | 2010 | Ma (40º33’N 3º32’E) | Spain | L | JX089502 |
|  | UMB-584.10 | 2010 | Ma (40º33’N 3º32’E) | Spain | L | JX089503 |
|  | UMB-585.10 | 2010 | Ma (40º33’N 3º32’E) | Spain | L | JX089504 |
|  | UMB-586.10 | 2010 | Ma (40º33’N 3º32’E) | Spain | L | JX089505 |
|  | UMB-587.10 | 2010 | Ma (40º33’N 3º32’E) | Spain | L | JX089506 |
|  | UMB-588.10 | 2010 | Ma (40º33’N 3º32’E) | Spain | L | JX089507 |
|  | UMB-607.10 | 2010 | Ma (40º33’N 3º32’E) | Spain | L | JX089508 |
|  | UMB-795.11 | 2011 | On (41º98’N 2º81’E) | Spain | L | JX089509 |
|  | UMB-797.11 | 2011 | On (41º98’N 2º81’E) | Spain | L | JX089510 |
|  | UMB-799.11 | 2011 | On (41º98’N 2º81’E) | Spain | L | JX089511 |
|  | UMB-800.11 | 2011 | On (41º98’N 2º81’E) | Spain | L | JX089512 |
| *Geniculospora grandis* (Greath.) Nolan | | | | | | |
|  | UMB-723.11 | 2011 | NN2 (30º22’S 152º54’E) | Australia | L | JX089513 |
|  | UMB-806.11 | 2011 | NN2 (30º22’S 152º54’E) | Australia | L | JX089514 |
|  | UMB-807.11 | 2011 | NN2 (30º22’S 152º54’E) | Australia | L | JX089515 |
|  | UMB-808.11 | 2011 | NN2 (30º22’S 152º54’E) | Australia | L | JX089516 |
|  | UMB-809.11 | 2011 | NN2 (30º22’S 152º54’E) | Australia | L | JX089517 |
|  | UMB-176.01 | 2001 | Lm (41°30’N 8°25’W) | Portugal | F | GQ411354^a^ |
|  | UMB-198.01 | 2001 | E1 (41°34’N 8°19’W) | Portugal | F | GQ411353^a^ |
|  | UMB-488.10 | 2010 | Vi (42º15N’ 8º71’W) | Spain | F | JX089518 |
|  | UMB-495.10 | 2010 | Vi (42º15’N 8º71’W) | Spain | F | JX089519 |
|  | UMB-502.10 | 2010 | Vi (42º15N’ 8º71’W) | Spain | F | JX089520 |
| *Lunulospora curvula* Ingold | | | | | | |
|  | UMB-659.10 | 2010 | NN1 (30º23’S 152º53’E) | Australia | L | JX089521 |
|  | UMB-666.10 | 2010 | Le (30º43’S 152º8’E) | Australia | L | JX089522 |
|  | UMB-670.10 | 2010 | Le (30º43’S 152º8’E) | Australia | L | JX089523 |
|  | UMB-688.10 | 2010 | Jo (30º14’S 152º5’E) | Australia | L | JX089524 |
|  | UMB-706.10 | 2010 | Le (30º43’S 152º8’E) | Australia | L | JX089525 |
|  | UMB-108.01 | 2001 | E5 (41°30’N 8°27’W) | Portugal | L | GQ411321^a^ |
|  | UMB-115.01 | 2001 | E5 (41°30’N 8°27’W) | Portugal | L | GQ411322^a^ |
|  | UMB-305.05 | 2005 | E1 (41°34’N 8°19’W) | Portugal | SW | GQ411320^a^ |
|  | UMB-498.09 | 2009 | Ve (41º99’N 8º33’W) | Portugal | L | JX089526 |
|  | UMB-499.09 | 2009 | Ve (41º99’N 8º33’W) | Portugal | L | JX089527 |
|  | UMB-500.09 | 2009 | Ve (41º99’N 8º33’W) | Portugal | L | JX089528 |
|  | UMB-578.10 | 2010 | Ta (41º23’N 7º69’W) | Portugal | L | JX089529 |
|  | UMB-579.10 | 2010 | Ta (41º23’N 7º69’W) | Portugal | L | JX089530 |
|  | UMB-825.09 | 2009 | Ve (41º99’N 8º33’W) | Portugal | L | JX089531 |
|  | UMB-826.09 | 2009 | Ve (41º99’N 8º33’W) | Portugal | L | JX089532 |
|  | UMB-782.11 | 2011 | On (41º98’N 2º81’E) | Spain | L | JX089533 |
|  | UMB-783.11 | 2011 | On (41º98’N 2º81’E) | Spain | L | JX089534 |
|  | UMB-791.11 | 2011 | On (41º98’N 2º81’E) | Spain | L | JX089535 |
| *Tetrachaetum elegans* Ingold | | | | | | |
|  | UMB-648.10 | 2010 | Ho (30º14’S 153º9’E) | Australia | L | JX089536 |
|  | UMB-810.11 | 2011 | Ho (30º14’S 153º9’E) | Australia | L | JX089537 |
|  | UMB-811.11 | 2011 | Ho (30º14’S 153º9’E) | Australia | L | JX089538 |
|  | UMB-812.11 | 2011 | Ho (30º14’S 153º9’E) | Australia | L | JX089539 |
|  | UMB-813.11 | 2011 | Ho (30º14’S 153º9’E) | Australia | L | JX089540 |
|  | UMB-435.09 | 2009 | Ve (41º99’N 8º33’W) | Portugal | L | JX089541 |
|  | UMB-483.09 | 2009 | Ca (41°38’N 8°19’W) | Portugal | L | JX089542 |
|  | UMB-522.10 | 2010 | Can (40º08’N 8º20’W) | Portugal | L | JX089543 |
|  | UMB-529.10 | 2010 | Can (40º08’N 8º20’W) | Portugal | L | JX089544 |
|  | UMB-532.10 | 2010 | Can (40º08’N 8º20’W) | Portugal | L | JX089545 |
|  | UMB-534.10 | 2010 | Can (40º08’N 8º20’W) | Portugal | L | JX089546 |
|  | UMB-609.10 | 2010 | Pet (41º48’N 8º01’W) | Portugal | L | JX089547 |
|  | UMB-615.10 | 2010 | Pet (41º48’N 8º01’W) | Portugal | L | JX089548 |
|  | UMB-617.10 | 2010 | Pet (41º48’N 8º01’W) | Portugal | L | JX089549 |
|  | UMB-717.11 | 2011 | Can (40º08’N 8º20’W) | Portugal | L | JX089550 |
| *Tricladium chaetocladium* Ingold | | | | | | |
|  | UMB-631.10 | 2010 | Ho (30º14’S 153º9’E) | Australia | L | JX089551 |
|  | UMB-642.10 | 2010 | Jo (30º14’S 152º5’E) | Australia | L | JX089552 |
|  | UMB-644.10 | 2010 | Jo (30º14’S 152º5’E) | Australia | L | JX089553 |
|  | UMB-667.10 | 2010 | Le (30º43’S 152º8’E) | Australia | L | JX089554 |
|  | UMB-685.10 | 2010 | Jo (30º14’S 152º5’E) | Australia | L | JX089555 |
|  | UMB-686.10 | 2010 | Jo (30º14’S 152º5’E) | Australia | L | JX089556 |
|  | UMB-687.10 | 2010 | Jo (30º14’S 152º5’E) | Australia | L | JX089557 |
|  | UMB-689.10 | 2010 | Jo (30º14’S 152º5’E) | Australia | L | JX089558 |
|  | UMB-690.10 | 2010 | Jo (30º14’S 152º5’E) | Australia | L | JX089559 |
|  | UMB-691.10 | 2010 | Jo (30º14’S 152º5’E) | Australia | L | JX089560 |
|  | UMB-711.10 | 2010 | Jo (30º14’S 152º5’E) | Australia | L | JX089561 |
|  | UMB-013.99 | 1999 | L1 (41°33’N 8°14’W) | Portugal | L | GQ411309^a^ |
|  | UMB-035.01 | 2001 | E5 (41°30’N 8°27’W) | Portugal | L | GQ411310^a^ |
|  | UMB-062.01 | 2001 | E5 (41°30’N 8°27’W) | Portugal | L | GQ411308^a^ |
|  | UMB-065.01 | 2001 | Pel (41°24’N 8°30’W) | Portugal | L | GQ411307^a^ |
|  | UMB-523.10 | 2010 | Can (40º08’N 8º20’W) | Portugal | L | JX089562 |
|  | UMB-527.10 | 2010 | Can (40º08’N 8º20’W) | Portugal | L | JX089563 |
|  | UMB-547.10 | 2010 | Ta (41º23’N 7º69’W) | Portugal | L | JX089564 |
|  | UMB-549.10 | 2010 | Ta (41º23’N 7º69’W) | Portugal | L | JX089565 |
|  | UMB-616.10 | 2010 | Pet (41º48’N 8º01’W) | Portugal | L | JX089566 |
|  | UMB-618.10 | 2010 | Pet (41º48’N 8º01’W) | Portugal | L | JX089567 |
|  | UMB-716.11 | 2011 | Can (40º08’N 8º20’W) | Portugal | L | JX089568 |
|  | UMB-720.11 | 2011 | Can (40º08’N 8º20’W) | Portugal | L | JX089569 |
|  | UMB-721.11 | 2011 | Can (40º08’N 8º20’W) | Portugal | L | JX089570 |
|  | UMB-504.10 | 2010 | Vi (42º15’N 8º71’W) | Spain | F | JX089571 |
|  | UMB-516.10 | 2010 | Vi (42º15’N 8º71’W) | Spain | F | JX089572 |

^a^, sequences retrieved from NCBI
